# Body composition and renal cell carcinoma prognosis in elderly patients: a retrospective cohort study

**DOI:** 10.1186/s12894-026-02149-7

**Published:** 2026-04-17

**Authors:** Heini Pajunen, Thea Veitonmäki, Jonne Åkerla, Akseli Aittoniemi, Joonas Ronkainen, Heini Huhtala, Jussi Nikkola, Teemu J. Murtola, Irina Rinta-Kiikka

**Affiliations:** 1 Department of Urology, TAYS Cancer Center, Tampere, Finland; 2 Faculty of Medicine and Health Technology, Tampere University, Tampere, Finland; 3 Department of Radiology, Tampere University hospital, Tampere, Finland; 4 Faculty of Social Sciences, Tampere University, Tampere, Finland

**Keywords:** Renal cell carcinoma, Body composition, Elderly

## Abstract

**Background:**

Body composition has been proposed as a prognostic factor in renal cell carcinoma (RCC), but evidence remains inconsistent, particularly in elderly patients. This study aimed to evaluate whether computed tomography (CT)-derived body composition indices are associated with overall survival (OS) in patients aged ≥ 75 years with RCC.

**Methods:**

This retrospective cohort study included all RCC patients aged ≥ 75 years diagnosed at Tampere University Hospital between 2003 and 2017 with available abdominal CT imaging.The study cohort comprised 239 patients. The formation of the dataset is illustrated in Figure 1. Body composition parameters, including psoas muscle index (PMI), skeletal muscle index (SMI), visceral adipose tissue index (VATI), subcutaneous adipose tissue index (SATI), and waist circumference, were measured at the third lumbar vertebra level. The primary outcome was OS, and the secondary outcome was risk of surgical complications. Cox proportional hazards models were used for survival analyses, with adjustment for established prognostic factors. Subgroup analyses were performed, and associations with surgical outcomes were assessed using correlation and logistic regression analyses.

**Results:**

A total of 239 patients aged ≥ 75 years with RCC were included. Median age at diagnosis was 80 years (75–94), and 67% underwent surgical treatment. Median follow-up was 4 (0-21) years. Sarcopenia was common across the cohort as low muscle mass was found in 95–100% of women and 94–98% of men. In univariable analysis, waist circumference showed a weak inverse association with OS (HR 0.99, 95% confidence interval (CI): 0.98–1.00). In subgroup analysis higher VATI was associated with improved survival among non-operated subgroup (HR 0.43, 95% CI 0.19–0.93). However, these findings were inconsistent and of small magnitude. No significant associations were observed in sex-stratified analyses. For surgical outcomes, weak correlations were found between waist circumference and VATI with intraoperative blood loss (*r* = 0.24, *p* = 0.024), SATI with length of hospital stay (*r* = 0.22, *p* = 0.013), and VATI with intraoperative organ injury (OR 1.01, 95% CI 1.00–1.02). After correction for multiple testing, only the association between VATI and blood loss remained significant. Overall, effect sizes were small.

**Conclusion:**

CT-derived body composition indices do not appear to have clinically meaningful utility in predicting OS or surgical complications in elderly RCC patients. These findings suggest that body composition measurements derived from routine CT imaging should not be used in isolation for risk stratification in this population. Instead, treatment decisions for elderly RCC patients should be based on a comprehensive geriatric assessment.

**Supplementary Information:**

The online version contains supplementary material available at 10.1186/s12894-026-02149-7.

## Background

Renal cell carcinoma (RCC) is a major contributor to cancer-related deaths globally, with clear cell RCC being the predominant histological subtype [1, 2]. In 2021, there were 1,037 new cases of RCC and 340 deaths due to RCC in Finland [3]. While RCC typically occurs in individuals aged 50–70, its prevalence is particularly high in the elderly [2, 4]. Elderly RCC patients differ significantly from younger individuals in terms of treatment risks and outcomes. They more frequently present with comorbidities, polypharmacy, cognitive decline, and sarcopenia, all of which complicate surgical decision-making and increase the risk of postoperative complications, mortality, and institutionalization [5–7].

Body composition has been identified as an important prognostic factor in many chronic and malignant diseases, especially in the elderly [8]. Although body mass index (BMI) is commonly used as a measure of obesity, it has limitations in assessing specific tissue quantities, particularly differences in fat distribution between visceral adipose tissue (VAT) and subcutaneous adipose tissue (SAT), and in distinguishing between different fat and skeletal muscle compartments [9, 10]. In addition, in older adults BMI does not account for body composition [11]. As a result, patients with the same BMI can have significantly different amounts of skeletal muscle and adipose tissue [12].

Imaging techniques like computed tomography (CT) are increasingly being used to accurately measure and differentiate between various abdominal fat deposits and muscle compartments in studies of body composition [9].

Cancer cachexia, a condition marked by the progressive loss of skeletal muscle and sometimes fat, is linked to a significant decrease in survival rates and quality of life [9]. Cachexia and malignancy-related weight loss have long been recognized as factors affecting cancer prognosis and treatment response [1, 13]. Alongside cachexia, physiological sarcopenia often develops, referring to a disorder of the skeletal muscles characterized by progressive decreases in muscle mass and strength [14]. Normally muscle tissue undergoes continuous growth and remodeling, but in sarcopenia, the balance between protein anabolism and catabolism changes, leading to muscle mass reduction [14]. Importantly, sarcopenia is considered a treatable condition and both pharmacological and non-pharmacological treatment options are under active investigation [15, 16].

In RCC patients, sarcopenia has been found in up to 47% of those with localized disease and 29–68% of those with metastatic disease [17–19]. It has been suggested that the prevalence of low muscle mass in patients with RCC ranges from 43% to 44%^20^. Some studies have found sarcopenia to be associated with poorer prognosis, while others have not found a statistical link between sarcopenia and RCC prognosis [17, 18, 21, 22]. Previous studies have shown that reduced skeletal muscle mass is an independent predictor of poorer overall survival (OS) in both localized and metastatic RCC [20].

Diagnosing sarcopenia typically requires measurement of muscle mass, strength, and physical performance. CT scan, routinely performed in cancer staging, provides the most accurate method to quantify muscle and fat tissue [23, 24]. Measuring the cross-sectional area of the psoas muscle and the abdominal wall muscles at the level of the third lumbar vertebra (L3) is a widely used method for assessing muscle mass. The resulting psoas muscle index (PMI) and skeletal muscle index (SMI) are commonly used indicators of sarcopenia and have been associated with patient prognosis [25–27].

Nephron-sparing surgery is the standard of care for patients with small RCC whenever feasible. Adherent perinephric fat (APF) can limit mobilization of the kidney and isolation of the renal tumor, potentially complicating the operation or increasing the risk of complications [28]. Khene et al. reported that APF was associated with malignant renal histology and adverse perioperative outcomes, including longer operative time and increased blood loss [29].

Obesity raises RCC risk, yet obese patients show lower mortality than normal-weight patients—a pattern called the obesity paradox [30]. In RCC, higher BMI often predicts better OS, possibly due to effects on fatty-acid metabolism, angiogenesis, and peritumoral inflammation, while lower BMI is linked to better surgical metrics such as shorter procedure and warm ischemia times [11, 31, 32]. Previous studies have shown that obesity and an increased amount of visceral fat are associated with greater surgical difficulty and a higher risk of postoperative complications in both radical and partial nephrectomy [33]. Older patients and those with comorbidities have also been found to have a higher risk of surgical complications [34].

Body composition and its potential associations with OS and complications are particularly important when planning treatment for elderly patients. However, existing research on the subject remains limited.

The aim of the study was to identify a simple, noninvasive CT-derived decision-making aid to measure body composition associated with outcomes in elderly RCC patients (≥ 75 years).

## Materials and methods

**Fig. 1:**
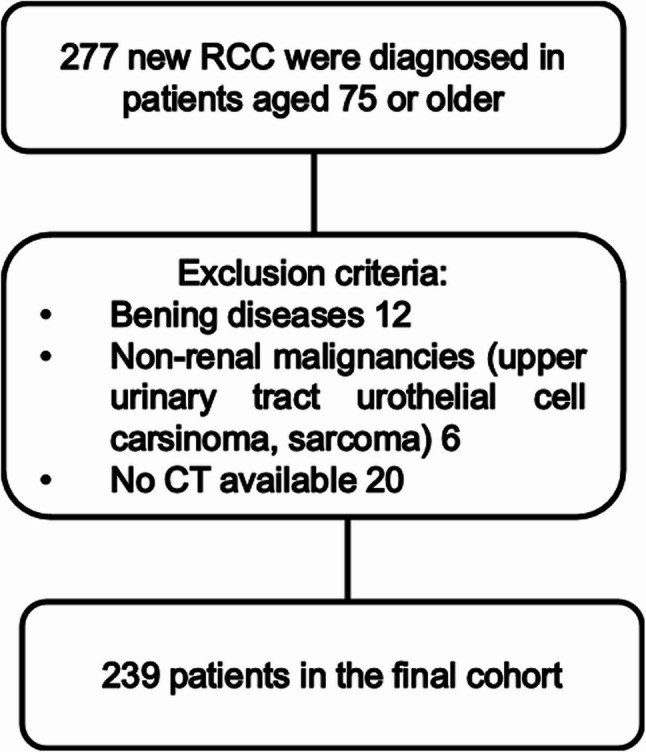
Patient selection flowchart

This is a retrospective registry study using data collected from the medical records of all new RCC patients diagnosed at Tampere University Hospital between 2003 and 2017. The formation of the dataset is illustrated in Fig. 1. Only cases with abdominal or whole-body CT scans were included. In addition to clinical data Charlson Comorbidity Index (CCI), metastatic disease, TNM, histology, operative treatment, intraoperative bleeding, need for intensive care, duration of hospital stay, Clavien-Dindo, OS, transition to palliative care, damage to other organs during surgery, and BMI, and body composition measurements (PMI, SMI, visceral adipose tissue index (VATI), subcutaneous adipose tissue index (SATI), waist circumference, and perinephric fat thickness) were taken using a single CT scan image at the middle level of L3.

Body composition parameters were quantified using 3D Slicer 5.6.1 software based on standard density thresholds in Hounsfield Units (HU): − 29 to + 150 for skeletal muscle, -200 to -30 for VAT and SAT. The measurement method is illustrated in Supplementary Figs. 1 A and 1B. An experienced radiologist advised the researchers on the measurement protocol, verified the first 20 measurements, and subsequently performed random quality checks on one third of all measurements. Image assessors were blinded to patient survival data.

In cases of uncertainty – such as determining the exact mid-level of the L3 vertebrae – the radiologist performed all measurements personally. The radiologist’s and researchers’ results showed a high degree of agreement, confirming the reliability of the measurements. Slice volumes were divided by slice thickness to calculate the corresponding areas (cm²). Cross-sectional areas were then adjusted for height (m²) to derive the PMI (cm²/m²), SMI (cm²/m²), SATI (cm²/m²), and VATI (cm²/m²).

Waist circumference was measured from the CT images 2 cm above the umbilicus. The thickness of the perinephric fat tissue was measured bilaterally on axial CT images obtained at the level of the renal vein, using the technique described in previous studies [28, 35]. The method for perinephric fat measurement is presented in Supplementary Fig. 2.

Association between different body composition measures and RCC prognosis will be evaluated using 1, 5, and 10-year survival and mortality data. Muscle and adipose tissue indices were calculated for all patients with available data. In some cases, measurement was not possible – for example, due to fixation material obscuring the back muscles or, in obese patients, because subcutaneous fat extended beyond the field of view. Consequently, the number of patients for whom muscle and fat indices could be calculated varies slightly.

### Statistical analysis

The primary outcome was OS, and the secondary outcome was the risk of surgical complications. Information on mortality was obtained from patient records. Follow-up began at the time of RCC diagnosis on CT between 2003 and 2017 and continued until death, loss to follow-up, or common closing date in May 2025.

Univariable Cox regression analyses were performed for each body composition index to assess the predictive value of PMI, SMI, VATI, SATI, and waist circumference for OS. In the multivariable analyses, the Cox regression model was adjusted for sex, age, CCI, presence of metastasis at diagnosis, symptoms, PMI, SMI, VATI, SATI, waist circumference and TNM stage. The final multivariable model included established prognostic factors associated with RCC mortality. The proportional hazards assumption was evaluated using Schoenfeld residuals.

Additionally, survival analyses were performed in predefined subgroups (sex; surgery vs. no surgery; and cytoreductive vs. curative vs. delayed surgery vs. inoperable disease) to evaluate potential differences across clinical scenarios.

As an exploratory analysis, survival analyses were repeated using body composition indices categorized as binary variables, comparing the highest quartile with the three lower quartiles. Additionally, non-parametric correlation analysis (Spearman) was used to assess associations between body composition indices and intraoperative blood loss, postoperative complications (Clavien–Dindo classification), need for intensive care, and length of hospital stay after surgery. To account for multiple comparisons, Bonferroni correction was applied. Finally, logistic regression analysis (forward likelihood ratio method) was performed to evaluate the association between body composition indices and intraoperative injury to other organs.

All statistical analyses were performed using SPSS version 29.

## Results

### Study cohort characteristics

Patients’ characteristics are presented in Table 1 and radiological characteristics in Table 2. A total of 239 RCC patients aged 75 years or older were included in the final cohort. Median follow-up was 4 (0–21) years. Median follow-up was 5 (0.08-21) years in the operated group and 1 (0–16) year in the non-operated group.

**Table 1:** Patient characteristics

Characteristics	All				Male				Female		
	*n* (%)	Mean	SD		*n* (%)	Mean	SD		*n* (%)	Mean	SD
Patients	239 (100)				102 (43)				137 (57)		
Age (years)	239 (100)	80.2	4.0		102 (43)	79.8	3.9		137 (57)	80.6	4.1
BMI (kg/m²)	200 (84)	26.6	5.6		91 (89)	26.3	4.3		137 (57)	21.4	12.3
< 25	89 (45)				37 (36)				80 (58)		
25–30	64 (32)				36 (35)				28 (20)		
> 30	47 (24)				18 (18)				29 (21)		
CCI	239 (100)	5.5	2.3		102 (100)	5.7	2.3		137 (100)	5.4	2.3
Solitary tumor	227 (95)				97 (95)				130 (95)		
Metastasis at diagnosis	239 (100)				102 (100)				137 (100)		
Yes	71 (30)				30 (29)				41(29)		
No	168 (71)				72 (71)				96 (70)		
Symptoms	155 (65)				68 (67)				87 (64)		
Operative treatment	161 (67)				72 (71)				89 (65)		
Nephrectomy	140 (87)				64 (89)				76 (85)		
Partial nephrectomy	21 (13)				8 (11)				13 (15)		
Cytoreductive operation	24 (10)				12 (12)				12 (9)		
Laparoscopic approach	54 (23)				26 (26)				28 (20)		
Open surgery	107 (45)				46 (45)				61 (45)		
Need for intraoperative blood transfusion	44 (27)				20 (27)				24 (27)		
Need for intensive care	12 (7)				5 (7)				7 (8)		
Clavien-Dindo 0–2	78 (48)				37 (51)				41 (46)		
Clavien-Dindo ≥ 3	13 (8)				4 (6)				9 (10)		
Non-operatively treated	78 (33)				30 (29)				48 (35)		
Survival	239 (100)				102 (100)				137 (100)		
Alive at 1 year	175 (73)				74 (73)				101 (74)		
Alive at 5 years	93 (39)				39 (38)				54 (40)		
Alive at 10 years	22 (9)				10 (10)				12 (9)		
Palliative care	100 (42)				41 (40)				59 (43)		

*BMI* Body mass index, *CCI* Charlson comorbidity index

**Table 2:** Radiological characteristics

Characteristics	All				Male				Female		
	*n* (%)	Mean	SD		*n* (%)	Mean	SD		*n* (%)	Mean	SD
PMI	192 (80)	3.5	1.9		87 (85)	3.6	1.6		105 (76)	3.4	2.1
SMI	192 (80)	21.2	10.3		87 (85)	23.1	10.2		105 (76)	19.7	10.1
VATI	193 (81)	62.8	54.0		87 (85)	68.8	43.1		106 (77)	57.8	61.3
SATI	191 (80)	61.1	45.3		86 (84)	45.3	31.0		105 (76)	74.0	50.8
Waist circumference (cm)	233 (98)	100.3	12.9		99 (97)	99.1	11.4		134 (97)	101.2	13.9
Perinephric fat thickness (mm)	234 (98)	25.5	11.1		99 (97)	30.7	10.5		135 (98)	21.6	9.9
< 1.0	13 (6)				0 (0)				0 (0)		
1.0-1.9	59 (25)				13 (13)				0 (0)		
≥ 2.0	162 (69)				86 (87)				76 (100)		
Tumor size (mm)	239 (100)	67.9	34.3		102 (100)	72.3	36.6		137 (100)	64.6	32.2
TNM	239 (100)				102 (100)				137 (100)		
pT1	81 (34)				27 (27)				54 (39)		
pT2	20 (8)				10 (10)				10 (7)		
pT3	117 (49)				54 (53)				63 (46)		
pT4	21 (9)				11 (11)				10 (7)		

*PMI* Psoas muscle index, *SMI* Skeletal muscle index, *VATI* Visceral adipose tissue index, *SATI* Subcutaneous adipose tissue index, *TNM* Tumor, nodes and metastasis classification

The median age at diagnosis was 80 (75–94) years. Although RCC is generally more common in men, women comprised the majority in this elderly cohort (*n* = 137, 57%) compared to men (*n* = 102, 43%). The median BMI was 25.7 (16.7–46.9). Sarcopenia was defined based on low muscle mass according to the previously described protocol [20]. Among men, 94% had low muscle mass according to the PMI and 98% according to the SMI. The corresponding proportions in women were 100% and 95%, respectively.

The median CCI was 5 (2–13). The median tumor size was 68 (10–170) mm. According to the TNM classification, the most common tumor stage was T3, observed in 117 cases (49%). At diagnosis, symptoms included fatigue, pain, weight loss, and abdominal resistance. Clear cell carcinoma was the most common histological subtype, identified in 145 patients (61%). The median length of hospital stay following surgery was 6 (1–16) days.

### Overall survival

In univariable Cox regression analysis, waist circumference showed a weak statistical inverse association with OS (HR 0.99, 95% CI: 0.98–1.00), suggesting a marginally lower risk of death with increasing waist circumference. However, this association was not statistically significant in the multivariable analysis, indicating limited clinical relevance. The results of the univariable and multivariable analyses are presented in Table 3.

**Table 3:** Risk of death in univariable and multivariable analyses

	*N* Cases/Deaths	HR Univariate (95% Cl)	HR Multivariate (95% Cl) *
PMI	192/152	0.97 (0.89–1.06)	0.98 (0.89–1.07)
SMI	192/152	0.99 (0.98–1.01)	1.00 (0.98–1.01)
VATI	193/152	1.00 (1.00–1.00)	1.00 (1.00–1.00)
SATI	191/150	1.00 (1.00–1.00)	1.00 (1.00–1.00)
Waist circumference	233/190	0.99 (0.98-1.00)	0.99 (0.98-1.00)

*HR* Hazard ratio, *Cl* Confidence interval, *PMI* Psoas muscle index, *SMI* Skeletal muscle inde, *VATI* Visceral adipose tissue index, *SATI* Subcutaneous adipose tissue index

* Calculated with proportional hazards degression with model adjustment for CCI, age, year of diagnosis, sex, TNM, symptoms, metastasis at diagnosis

**Fig. 2:**
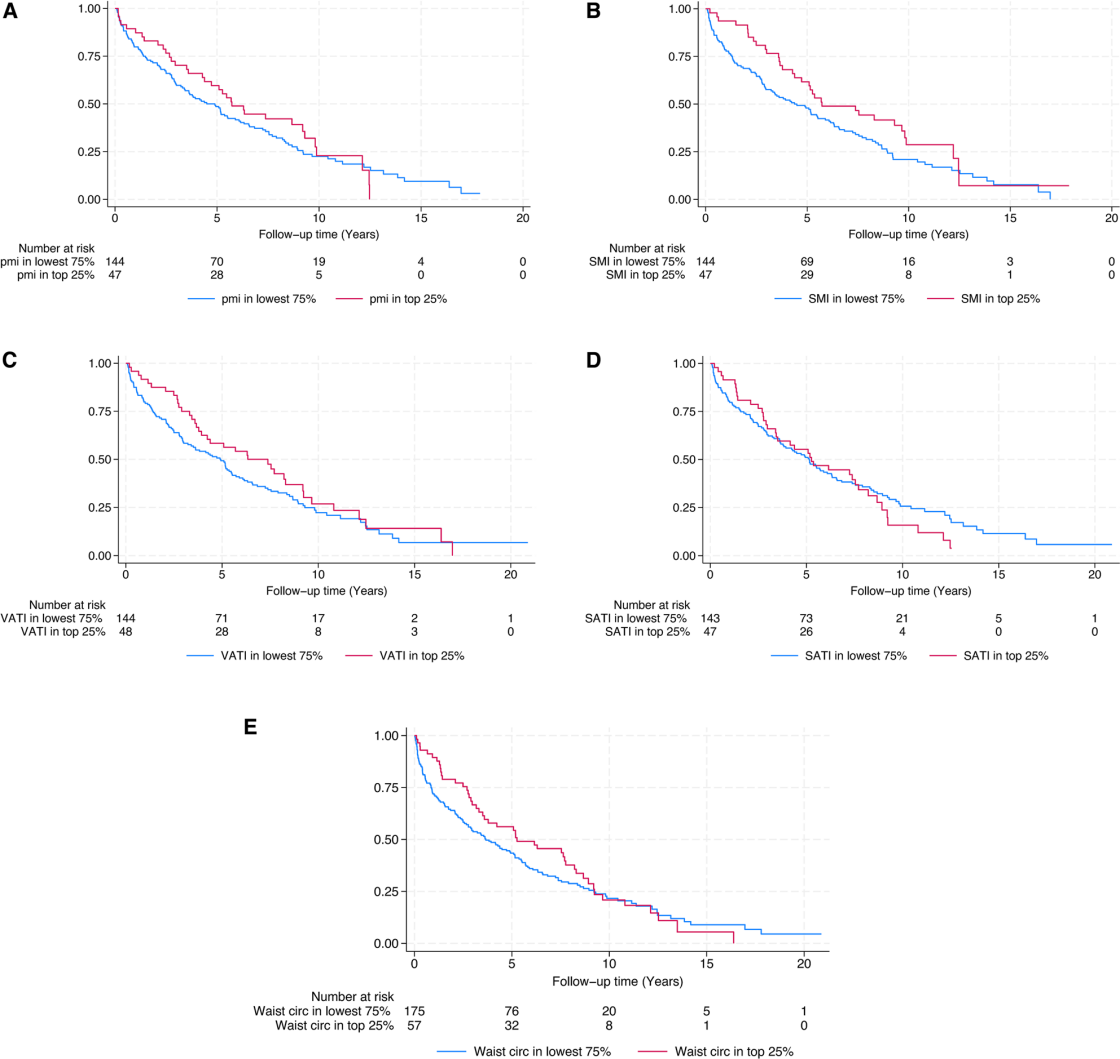
Kaplan–Meier curves for muscle and fat indices and waist circumference. Figure legend: The comparison is made using dichotomized values, where the top quartile is compared to the three bottom quartiles. Figure 2 **A** presents PMI, Fig. 2**B** SMI, Fig. 2**C** VATI, Fig. 2**D** SATI, and Fig. 2**E** waist circumference

To assess the impact of missing data, a complete case analysis restricted to cases with available height and BMI data was performed, with no effect on the results. These findings are presented in Supplementary Table 1. Kaplan–Meier curves for muscle and fat indices and waist circumference are shown in Figs. 2A–E.

In subgroup analysis of non-operated patients, waist circumference remained a borderline significant predictor of OS in both univariable (HR 0.98, 95% CI: 0.96–0.99) and multivariable analyses (HR 0.98, 95% CI: 0.96–1.00).

When radiological variables were dichotomized (lowest 75% vs. highest 25%), no significant associations with OS were observed in either univariable or multivariable Cox regression analyses. Dichotomization by median values is presented in Supplementary Table 2. In subgroup analysis, univariable models indicated that higher SMI among operated patients (HR 0.65, 95% CI: 0.42–1.00) and higher waist circumference among non-operated patients (HR 0.52, 95% CI: 0.28–0.96) were associated with improved survival. In non-surgically managed patients, waist circumference remained of borderline significance (HR 0.54, 95% CI 0.30–1.01). In multivariable analysis, higher VATI was associated with improved survival among non-operated patients (HR 0.43, 95% CI 0.19–0.93).

Although some statistically significant associations were observed in subgroup analyses, these findings were inconsistent and of limited magnitude and are unlikely to be clinically meaningful. Overall, the results do not support a clinically relevant association between body composition indices and OS.

In sex-stratified subgroup analyses, no significant associations were observed in either univariable or multivariable models. Overall, the findings were more pronounced in the non-surgical subgroup.

### Risk of surgical complications

In non-parametric correlation analysis (Spearman), waist circumference and VATI showed weak correlations with intraoperative blood loss (correlation coefficients 0.31, *p* < 0.001 and 0.19, *p* = 0.024, respectively). Higher SATI was weakly correlated with longer postoperative hospital stay (*r* = 0.22, *p* = 0.013). No significant correlations were observed for length of intensive care unit (ICU) stay or Clavien–Dindo classification. After Bonferroni correction, only the association with VATI remained statistically significant.

In logistic regression analysis, higher VATI showed a weak association with intraoperative injury to adjacent organs (OR 1.01, 95% CI 1.00–1.02, per unit increase).

Despite a few statistically significant findings, the observed effect sizes were small and unlikely to be of clinical relevance. Overall, these results suggest no meaningful association between body composition indices and the risk of surgical complications.

## Discussion

Overall, our findings suggest that the clinical utility of body composition measurements derived from preoperative CT is limited in an elderly RCC population. This study evaluated the prognostic and surgical implications of radiological body composition indices—PMI, SMI, VATI, SATI, and waist circumference, in patients aged 75 years or older with RCC.

The most consistent finding was a weak inverse association between waist circumference and OS among non-surgically managed patients. In this subgroup, waist circumference remained a borderline predictor of improved survival, and higher VATI was also associated with better survival. These associations were primarily observed in the non-surgical subgroup, with no significant sex-specific differences.

Regarding surgical outcomes, waist circumference and VATI showed weak correlations with intraoperative blood loss, SATI with longer hospital stay, and VATI with intraoperative injury to adjacent organs. However, all observed associations were modest in magnitude.

Importantly, nearly all patients in this elderly cohort were classified as sarcopenic based on PMI or SMI (95–100% of women and 94–98% of men), underscoring the distinct body composition profile of older RCC patients.

Previous studies on the association between body composition and RCC prognosis have yielded inconsistent results [20, 36]. Wang et al. reported no associations between skeletal muscle or adipose tissue compartments and survival. Instead, a higher percentage of SAT independently predicted better progression-free survival and OS in patients with metastatic RCC [37]. Similarly, Mauriz et al. found that lower VATI was associated with worse OS in stage I-III disease in both sexes, while in stage IV diseases, higher VATI was associated with better OS in men. In women, the result was similar but not statistically significant [36].

Previous studies have attempted to determine the relationship between different fat distribution and OS in RCC, but the results have been contradictory [38]. Some studies suggest that moderate levels of adiposity may paradoxically be associated with improved survival in certain populations, a phenomenon often referred to as the “fat paradox.” Obesity may offer a survival advantage in RCC patients, possibly due to less aggressive tumors, greater metabolic reserves, or enhanced treatment response from increased inflammation, and immunogenicity [39]. Additional explanations for this paradox include better nutritional status, distinct gene expression, and molecular profiles observed in obese patients compared to those of normal weight [12].

Importantly, most prior studies have included younger populations with fewer comorbidities and better baseline functional status, which may partly explain discrepancies with the present findings in an exclusively elderly cohort.

Although CT imaging enables detailed assessment of body composition beyond tumor characteristics, the clinical relevance of these measures in elderly RCC patients appears limited. The observed inverse association between waist circumference and OS in non-surgically treated patients may reflect the “fat paradox”; however, the effect sizes were small and inconsistent.

Given that surgery remains the primary and most effective treatment for RCC, comprehensive preoperative assessment– including comorbidities, organ function, and performance status –remains essential. While CT-derived body composition metrics are readily available, their additional prognostic value in this elderly population appears minimal and unlikely to influence clinical decision-making.

Although some statistically significant associations were identified, these findings were inconsistent, of small magnitude, and unlikely to be clinically meaningful. Overall, our results suggest a null finding, with no robust or clinically relevant association between body composition indices and survival or surgical outcomes in elderly RCC patients.

In elderly populations, comprehensive geriatric assessment—including frailty, functional status, cognition, and nutritional evaluation—may provide more clinically relevant information than isolated radiological body composition measures.

This study has several limitations. Its retrospective design, missing data, and its single-center setting. CT images were obtained using varying protocols, and imaging practices have also changed slightly during the study. There were also differences in the administration and timing of the contrast agent, which may slightly affect the density measurements of the psoas muscle. Additionally, the section thickness varied depending on the year the CT scan was performed and the imaging device used. There may also be slight inaccuracies in the measurement of subcutaneous fat, as in particularly large individuals, part of the subcutaneous fat is cut off from the CT scan area and is not fully included in the calculation. The measurements were taken from retrospective CT images, where some of the subcutaneous fat might fall outside the imaging area. Furthermore, the study lacks external validation. These factors may limit the generalizability of the findings and should be considered when interpreting the results.

Selection bias is also likely, as patients undergoing surgery were generally fitter than those managed non-surgically. Furthermore, data on performance status, cognition, nutritional status, physical activity, and formal frailty assessments were not available, limiting the ability to fully account for treatment selection.

A key strength of this study is the inclusion of all patients aged 75 or older diagnosed with RCC at Tampere university hospital, providing a comprehensive real-world representation of this age group.

Future studies should utilize standardized RCC-specific CT protocols with consistent slice thickness and contrast timing to improve measurement reliability. Prospective, multicenter studies with external validation are needed to better define the prognostic value of body composition in elderly RCC populations.

Further research should also integrate radiological body composition metrics with comprehensive geriatric assessment, frailty indices, inflammatory markers, and molecular tumor profiling to better capture the complex interplay between aging, body composition, and cancer outcomes. Comparative studies including both younger and older RCC cohorts may help clarify the modifying effect of age and the role of potential selection biases.

## Conclusion

 CT-derived body composition indices do not appear to have clinically meaningful utility in predicting OS or surgical complications in elderly RCC patients. These findings suggest that body composition measurements derived from routine CT imaging should not be used in isolation for risk stratification in this population. Instead, treatment decisions for elderly RCC patients should be based on a comprehensive geriatric assessment.

## Supplementary Information

Supplementary Material 1: Supplementary Figs. 1 A. Measurements taken at the level of third lumbar vertebra (L3): Annotation example of visceral fat (brown) and subcutaneous fat (light blue).

Supplementary Material 2: Supplementary Figs. 1B. Measurements taken at the level of third lumbar vertebra (L3): Psoas muscle (light green) and skeletal muscle (yellow).

Supplementary Material 3: Supplementary Figs. 2. The fat thickness was measured in mm laterally (L) and posteriorly (P) to renal cortex at the level of the renal vein (RV).

Supplementary Material 4: Supplementary Table 1. Complete case analysis. Figure legend: PMI, psoas muscle index; SMI, skeletal muscle index; VATI, visceral adipose tissue index; SATI, subcutaneous adipose tissue index.

Supplementary Material 5: Supplementary Table 2. Body indices from Table 3 are dichotomized by medians. Figure legend: PMI, psoas muscle index; SMI, skeletal muscle index; VATI, visceral adipose tissue index; SATI, subcutaneous adipose tissue index.

## Data Availability

The data that support the findings of this study are available from the Wellbeing Services County of Pirkanmaa, but restrictions apply to the availability of these data, which were used under license for the current study, and so are not publicly available. Data are, however, available and can be requested from the Wellbeing Services County of Pirkanmaa.

## Abbreviations

RCC: Renal cell carcinoma
BMI: body mass index
VAT: visceral adipose tissue
SAT: subcutaneous adipose tissue
CT: computed tomography
OS: overall survival
L3: third lumbar vertebral
PMI: psoas muscle index
SMI: skeletal muscle index
APF: Adherent perinephric fat
CCI: Charlson Comorbidity Index
VATI: visceral adipose tissue index
SATI: subcutaneous adipose tissue index
HU: Hounsfield Units
RV: renal vein
L: lateral
P: posterior
HR: hazard ratio
CI: confidence interval
