# Supplementary figures and images for: Body composition and renal cell carcinoma prognosis in elderly patients: a retrospective cohort study
Source: BMC Urol. 2026 Apr 17;26:134. doi: 10.1186/s12894-026-02149-7 (PMC13231625; doi:10.1186/s12894-026-02149-7)

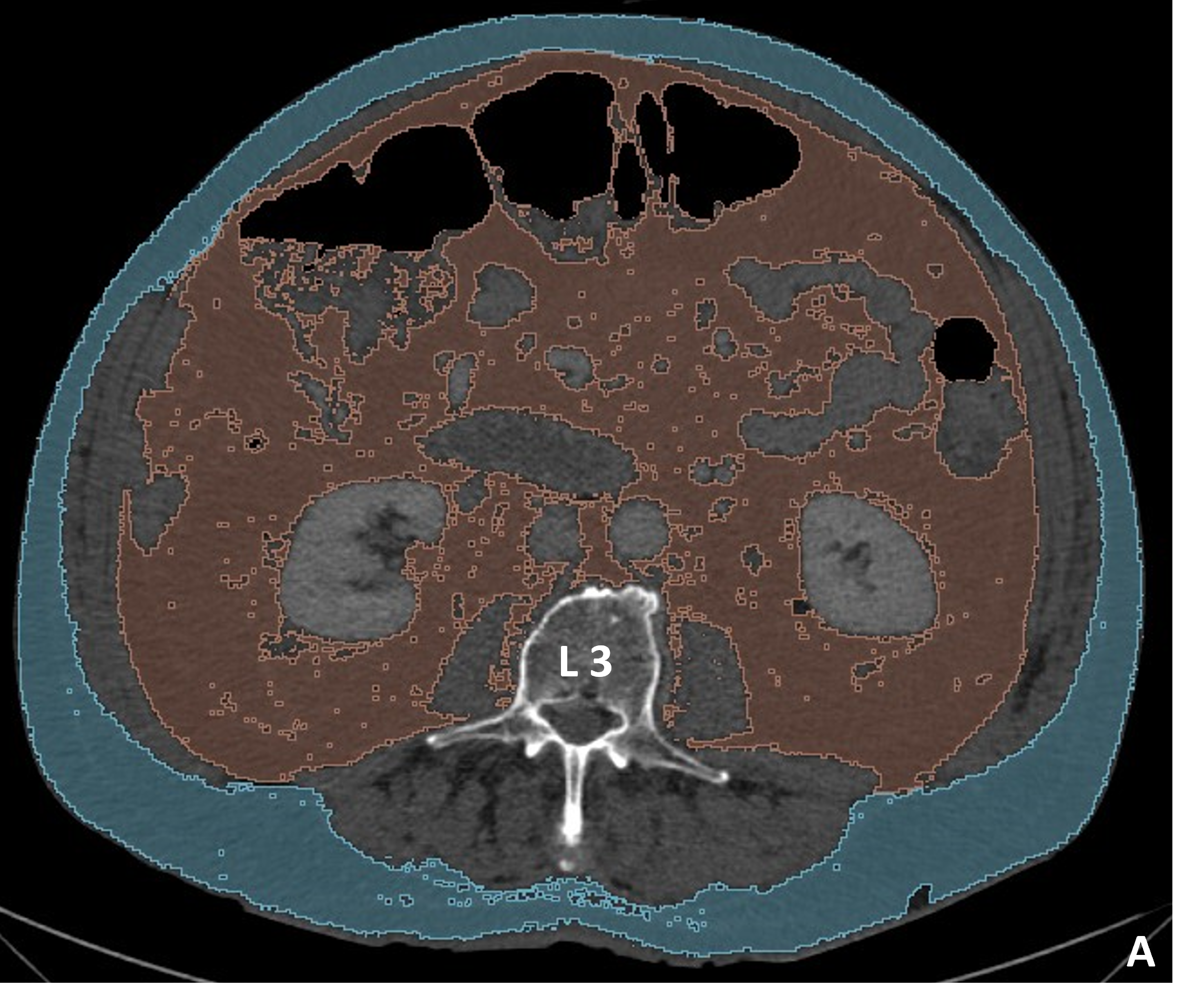

Supplement: Supplementary file 1 — Supplementary Material 1: Supplementary Figs. 1 A. Measurements taken at the level of third lumbar vertebra (L3): Annotation example of visceral fat (brown) and subcutaneous fat (light blue). [file 12894_2026_2149_MOESM1_ESM.tif]

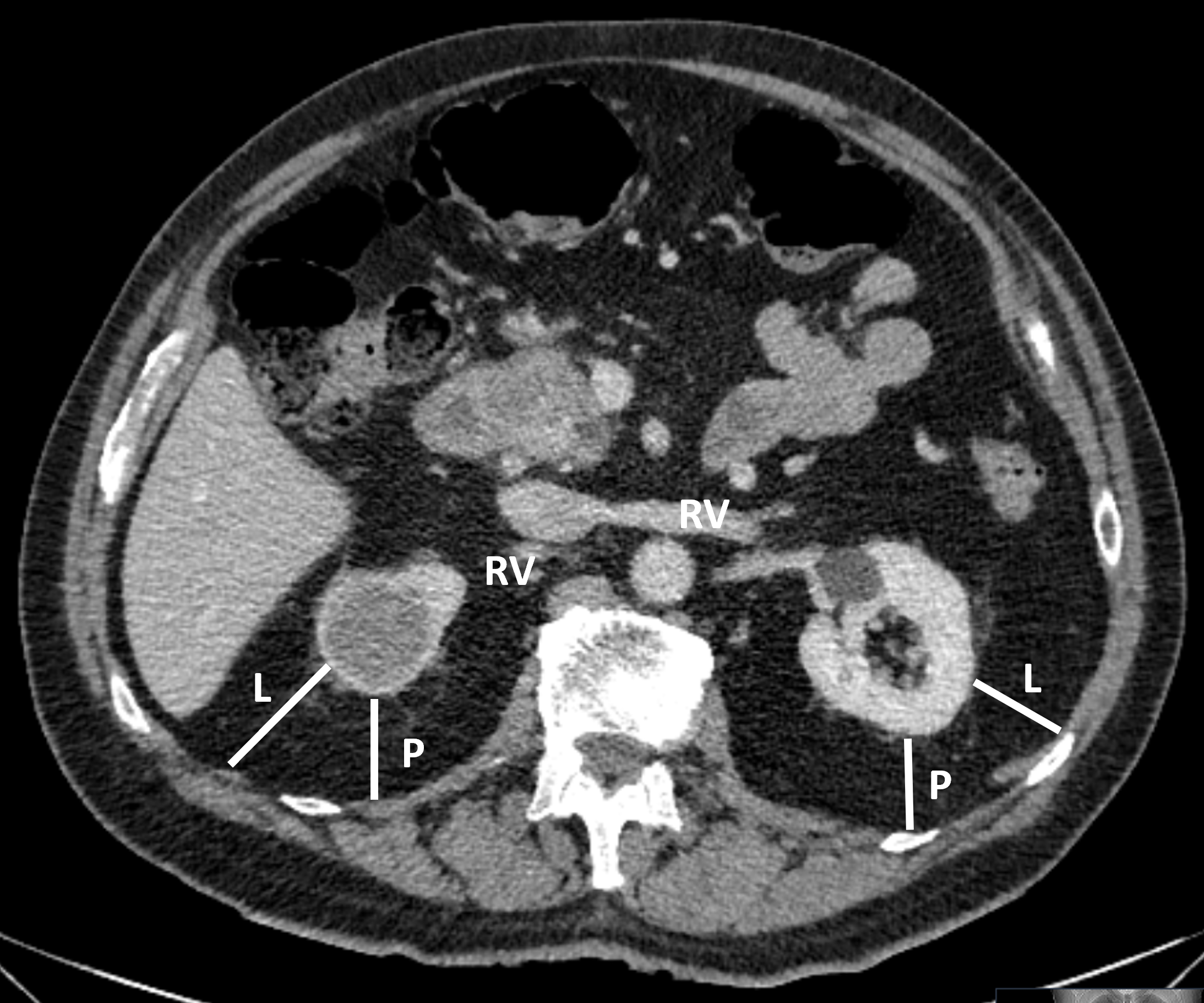

Supplement: Supplementary file 4 — Supplementary Material 4: Supplementary Table 1. Complete case analysis. Figure legend: PMI, psoas muscle index; SMI, skeletal muscle index; VATI, visceral adipose tissue index; SATI, subcutaneous adipose tissue index. [file 12894_2026_2149_MOESM4_ESM.tif]

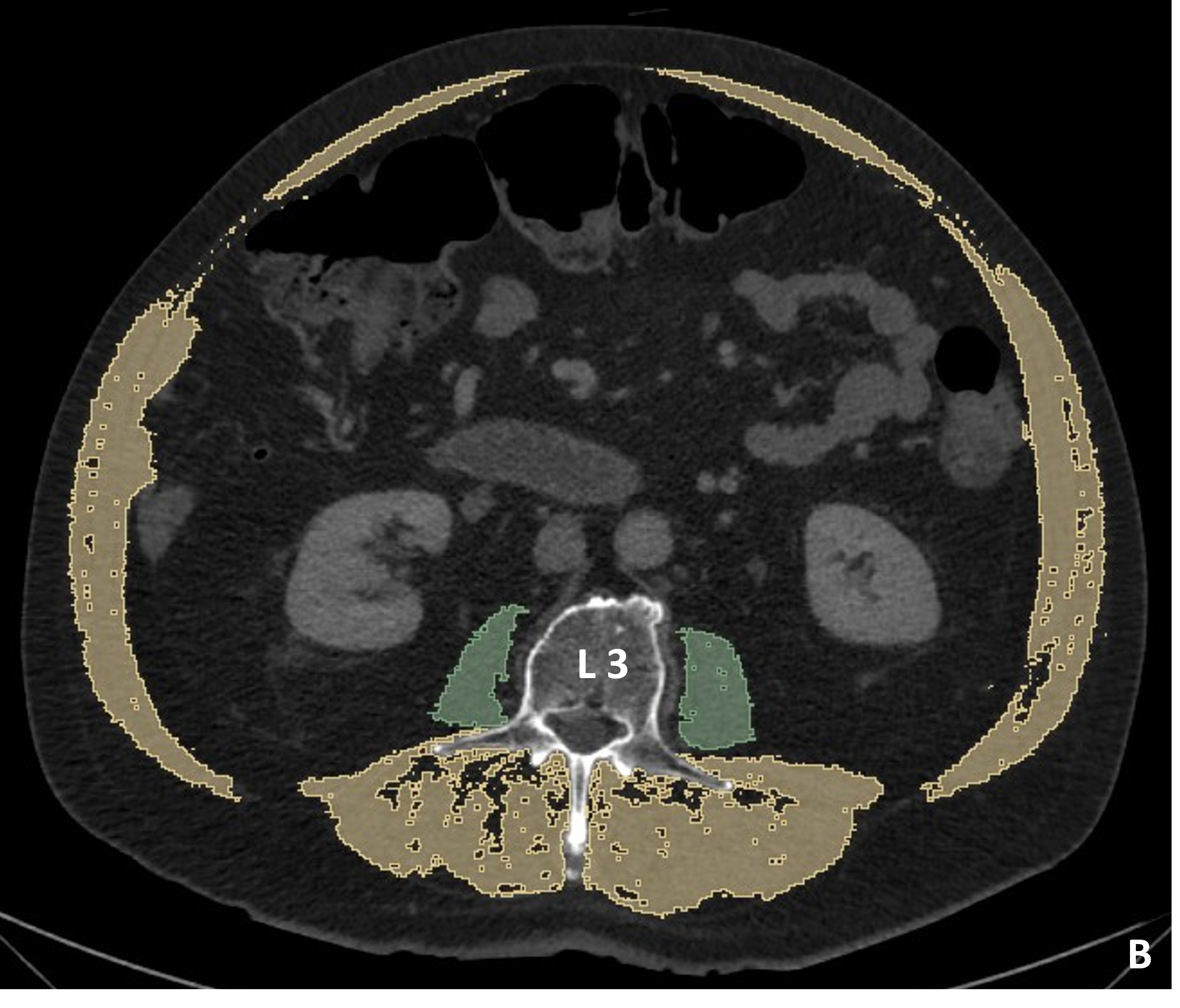

Supplement: Supplementary file 5 — Supplementary Material 5: Supplementary Table 2. Body indices from Table 3 are dichotomized by medians. Figure legend: PMI, psoas muscle index; SMI, skeletal muscle index; VATI, visceral adipose tissue index; SATI, subcutaneous adipose tissue index. [file 12894_2026_2149_MOESM5_ESM.tif]
