# Supplementary material for: Body composition and renal cell carcinoma prognosis in elderly patients: a retrospective cohort study
Source: BMC Urol. 2026 Apr 17;26:134. doi: 10.1186/s12894-026-02149-7 (PMC13231625; doi:10.1186/s12894-026-02149-7)
Supplement: Supplementary file 2 — Supplementary Material 2: Supplementary Figs. 1B. Measurements taken at the level of third lumbar vertebra (L3): Psoas muscle (light green) and skeletal muscle (yellow). [file 12894_2026_2149_MOESM2_ESM.docx]

|  | N Cases/Deaths | HR Univariate (95% Cl) | HR Multivariable (95% Cl) |
| --- | --- | --- | --- |
| PMI | 192/152 | 0.86 (0.63-1.19) | 0.91 (0.66-1.26) |
| SMI | 192/152 | 0.82 (0.60-1.13) | 0.90 (0.65-1.24) |
| VATI | 193/152 | 1.04 (0.75-1.43) | 0.96 (0.70-1.33) |
| SATI | 191/150 | 1.15 (0.83-1.58) | 1.08 (0.78-1.49) |
| Waist circumference | 233/190 | 0.96 (0.72-1.27) | 1.01 (0.76-1.35) |

PMI, psoas muscle index; SMI, skeletal muscle index; VATI, visceral adipose tissue index; SATI, subcutaneous adipose tissue index.
