# Supplementary material for: Body composition and renal cell carcinoma prognosis in elderly patients: a retrospective cohort study
Source: BMC Urol. 2026 Apr 17;26:134. doi: 10.1186/s12894-026-02149-7 (PMC13231625; doi:10.1186/s12894-026-02149-7)
Supplement: Supplementary file 3 — Supplementary Material 3: Supplementary Figs. 2. The fat thickness was measured in mm laterally (L) and posteriorly (P) to renal cortex at the level of the renal vein (RV). [file 12894_2026_2149_MOESM3_ESM.docx]

|  | N Cases/Deaths | HR Univariate (95% Cl) | HR Multivariable (95% Cl) |
| --- | --- | --- | --- |
| PMI | 140/40 | 0.98 (0.89-1.07) | 0.99 (0.90-1.08) |
| SMI | 140/40 | 0.99 (0.98-1.01) | 0.99 (0.98-1.01) |
| VATI | 140/40 | 1.00 (0.99-1.01) | 0.99 (0.98-1.00) |
| SATI | 140/40 | 1.00 (0.99-1.01) | 1.00 (0.99-1.01) |
| Waist circumference | 140/40 | 0.99 (0.98-1.00) | 0.99 (0.98-1.01) |

PMI, psoas muscle index; SMI, skeletal muscle index; VATI, visceral adipose tissue index; SATI, subcutaneous adipose tissue index.
